# Association between socioeconomic position of the household head, food insecurity and psychological health: an application of propensity score matching

**DOI:** 10.1186/s12889-024-20153-0

**Published:** 2024-09-27

**Authors:** Elias M. A. Militao, Olalekan A. Uthman, Elsa M. Salvador, Stig Vinberg, Gloria Macassa

**Affiliations:** 1 Department of Health Sciences, Faculty of Humanities, Mid Sweden University, Holmgatan 10, Sundsvall, SE-851 70 Sweden; 2 Department of Public Health and Sports Science, Faculty of Occupational and Health Sciences, University of Gävle, Kungsbacksvägen 47, Gävle, 80176 Sweden; 3 Department of Biological Sciences, Faculty of Science, Eduardo Mondlane University, 3453 Julius Nyerere Avenue, Maputo 257, Maputo, 257 Mozambique; 4 Warwick Centre for Global Health, Division of Health Sciences, Warwick Medical School, University of Warwick, Coventry, CV4 7AL UK; 5 Department of Global Health, Division of Epidemiology and Biostatistics, Faculty of Health Sciences, Stellenbosch University, Francie van Zijl Drive, Cape Town, 7505 South Africa; 6 Department of Public Health, School of Health Sciences, University of Skövde, Skövde, 541 28 Sweden; 7 EPI Unit, Instituto de Saúde Pública, Universidade do Porto, Rua das Taipas 135, Porto, 4050-600 Portugal

**Keywords:** Socioeconomic position, Food insecurity, Psychological health outcomes, Anxiety and depression, Heads of household

## Abstract

**Background:**

Mental health outcomes can be influenced by various factors, one of which has recently gained attention, namely food security. Food security is paramount to maintaining not only physical, but also mental health. There is an increasing need to understand the interplay between food insecurity (FI) and mental health outcomes, especially among vulnerable populations. The objective of this study was to investigate the effect of FI on psychological health (anxiety and depression) as well as to examine the modifying effect of socioeconomic position on this relationship.

**Methods:**

A cross-sectional study was conducted in Maputo City, Mozambique, in 1,842 participants. Data were collected through structured interviews using a modified version of the US Department of Agriculture Household Food Security Module to measure FI, and the Hospital Anxiety and Depression Scale to measure anxiety and depression. A composite variable for psychological health was created. Propensity score matching and interaction effect analyses were employed to examine the effects of FI on psychological health and the moderating role of socioeconomic position.

**Results:**

Of the 1,174 participants randomly assigned to propensity score matching, 787 were exposed to FI while 387 were unexposed. The analysis revealed stark disparities in psychological health outcomes associated with FI. The risk of poor psychological health among those exposed to FI was 25.79%, which was significantly higher than the 0.26% in unexposed individuals. The risk difference was 25.54% points (95% CI: 22.44–28.63), with a risk ratio of 99.82. Our assessment of population attributable fractions indicated that nearly all the risk for poor psychological health in the exposed group could be ascribed to FI. The interaction effects analysis revealed that socioeconomic status modifies this relationship. Specifically, heads of food-insecure households with a lower socioeconomic position tended to report poor mental health compared to their food-secure counterparts with a higher position.

**Conclusions:**

The findings underscore the profound impact of FI on the mental health of household heads in Maputo City, socioeconomic position being a significant modifier. Addressing household FI along with the socioeconomic position of household heads could be pivotal to mental health promotion, especially among vulnerable populations.

**Supplementary Information:**

The online version contains supplementary material available at 10.1186/s12889-024-20153-0.

## Introduction

Despite global political and economic progress, many households continue to experience moderate and severe food insecurity (FI). Largely this is because of poverty and underemployment linked to various factors and inequalities (e.g. conflicts, governments and social protection systems, food systems) [1]. In fact, estimates indicate that not only has global extreme poverty increased, but so also has global income inequality, for the first time in 20 years [1].

One key aspect among food-insecure households is their inability to provide nutritionally adequate and safe foods for an active and healthy life [2]. Indeed, according to the Food and Agriculture Organization of the United Nations, food security (FS) exists when people always have physical, social, and economic access to sufficient, safe, and nutritious foods that meet their dietary needs and food preferences for an active and healthy life. On the other hand, FI exists when the ability to acquire nutritionally adequate and safe foods in socially acceptable ways is limited or uncertain. Therefore, FI is an ongoing public health issue across the world, although the situation is more complicated in low- and middle-income countries (LMICs) than in high-income countries (HICs) [1].

Today, mental disorders are recognized as significant contributors to the global burden of disease and as leading causes of years lived with disability (YLDs), accounting for 4.9% of all disability-adjusted life years (DALYs) [3]. Globally, almost one in three individuals experiences a common mental disorder (e.g. anxiety, depression, and somatic symptom disorder) during their lifetime, and FI is said to be a key contributor to common mental disorders [4]. Over 300 million people worldwide (4.4% of the global population) are estimated to suffer from major depression [5], and the same figure applies to anxiety [6]. Furthermore, these conditions have profound implications for the social and economic well-being of individuals, households and communities [7, 8]. Likewise, resources for mental health (e.g. infrastructure, funding, human resources, and essential medicines) are often very limited in LMICs including Mozambique [9, 10]. Additionally, because of the strong stigma attached to mental illness, notably in LMICs, the extent of the problem is often underestimated [11–13].

Recent research has shown a link between FI and mental disorders, especially stress, anxiety and depression, working through various pathways [4, 14, 15]. It is well documented that among food-insecure households, the quality of diet (food quality and safety) is highly compromised [16, 17]. This situation may lead individuals and households to acquire food in socially unacceptable ways [16, 17]. This induces feelings of alienation, helplessness, shame, and guilt associated with depression [4, 14]. Likewise, research evidence indicates that the relationship between household FI and emotional health can work in both ways [18], thus making these studies even more complex and demanding [19]. Food insecurity as a source of chronic stress can raise cortisol levels and cause hypothalamic-pituitary-adrenal dysfunction [20], which plays a key role in the development of affective disorders and depression [21, 22]. Poor diet is also associated with increased risk of cognitive decline [23, 24]. Stress and worry derived from FI can cause maladaptive responses, leading to suicidal ideation and behaviour. This along with other mental illnesses may lead to increased health expenses, unemployment, social withdrawal, and other effects which deteriorate each of these conditions [25].

Similarly, various studies have highlighted the impact of socioeconomic inequalities on household FI as well as mental health [26, 27]. In fact, inequalities in income, education and work are among the underlying causes of poverty and household FI, especially in LMICs [26], and are globally associated with increased risk of mental ill health [27]. For instance, poverty and low income can hinder access to nutritionally adequate and safe food among vulnerable populations [1]. Likewise, people experiencing poverty cannot afford adequate housing or quality health care, nor can they afford quality education for their families [28]. Education can shape food access and utilization [29], as well as making people more receptive to health services [28]. Additionally, it can provide better employment opportunities in terms of income and safety [29], and better jobs may provide social prestige and networks [28]. Conversely, when household heads are unemployed or underemployed, this can propel their families into poverty and FI, which in turn will contribute to and exacerbate mental health issues [30]. This situation is particularly important in the context of Mozambique, where the unemployment rate is about 18.4% [31] and over 80% of the population is engaged in informal labour [32].

On this topic, a study by Jones [4] including 149 countries found that FI was consistently associated with poorer mental health in a dose-response manner, even after controlling for potential socioeconomic confounders. The magnitude of this association was greater than that for socioeconomic variables (i.e. education, employment, household income). This reinforces the possible causal nature of this relationship and suggests that psychosocial stressors that trigger mental health problems can be amplified with increasing FI [4]. Similarly, in Africa, a systematic review by Trudell et al. [33] has demonstrated a dose-response relationship between FI and poorer mental health (anxiety, depression and/or other mental disorders). Various factors (e.g. age, sex, social support) were found to mediate this relationship, and the findings suggest that interventions designed to target livelihood and sustainability (e.g. employment, ownership of livestock) rather than just income might be more effective [33].

There are no national-level data quantifying the burden of mental illness and its associated factors in Mozambique, although the country’s suicide rate is the eighth highest in the world, at 23.2 per 100,000 population [34, 35]. One study, conducted by Audet et al. [34] in Zambézia Province (central Mozambique) among female heads of household, found that those experiencing FI had increased odds of depressive symptoms compared with their food-secure counterparts. Furthermore, high income, but not education, was found to have a protective effect against depression [34]. Altogether these findings suggest that FI plays an important role in shaping mental health outcomes, especially among vulnerable populations.

Although household FI has emerged as a key factor for mental health outcomes, there is limited information on the association of household FI and socioeconomic position (SEP) on mental health in the general population in Mozambique, and Maputo City in particular. Only a few studies from outside Africa have thus far explored how household FI and SEP interact to affect mental health [36–38]. Therefore, this study aimed to investigate the association between SEP, household FI and psychological ill health (anxiety and depression) of household heads in Maputo City.

## Methods

### Study setting and sampling

A cross-sectional study was conducted in Maputo, the capital city of Mozambique. The city is divided into seven municipal districts [39], and is the largest urban agglomeration in Mozambique. Traditionally, it is divided into three areas that include KaMpfumu district, the wealthiest area of the city; the suburbs of Nlhamankulu and KaMaxaquene; and the peri-urban districts of KaMavota and KaMubukwana [39]. In addition, there are the KaNyaka and Katembe districts. Maputo has about 65% of underemployment [40]. According to the 2017 general census, Maputo City has about 1,080,277 inhabitants and 235,750 households [41], and about 79% of households are food-insecure [42].

The selection of households in this study relied on a two-stage design inspired by Mozambique’s National Institute of Statistics. This was used by the Mozambique Technical Secretariat for Food Security and Nutrition (SETSAN) in their 2013 baseline study, as previously described by Militao et al. [42]. In the first stage, a total of 96 enumeration areas were randomly selected, where each area could provide a maximum of 20 households. Next, in the second stage, using a systematic random sampling strategy and based on approximate proportional allocation, a total of 1,842 households in four municipal districts (Nlhamankulu, KaMaxaquene, KaMavota and KaMubukwana) were selected.

### Data collection

Face-to-face structured interviews were carried out at each participant’s home between November 2021 and June 2022, just after somewhat strict measures to curb the spread of COVID-19. A questionnaire previously validated for Portuguese-speaking countries [43, 44] and adapted to the Mozambican context was used to collect data. The questionnaire contained eight items from the US Department of Agriculture Household Food Security Survey Module to measure FI in the last 3 months. The scale had a maximum of 8 points, and households were considered food secure if they scored ≤ 1, scores of 2 or 3 were considered mild FI, scores from 4 − 6 were moderate FI, and scores of 7 or 8 were considered severe FI. However, for study purposes, two group categories were applied (food secure vs. food insecure). Dietary patterns were assessed using a check list based on the variety of food items consumed in the last 7 days and how often they were consumed (at least in two meals), and for study purposes, three categories were created (low, medium, high) (for more details, see Militao et al. [42]). Additionally, the questionnaire included demographic and socioeconomic questions (education, work, income) as well as questions about: (a) physical health (diagnosed hypertension and type 2 diabetes); (b) mental health (self-reported anxiety and depression); (c) medication and health care utilization; (d) self-reported health; (e) sleeping patterns; (f) physical activity; and (g) the health behaviours (smoking, and use of alcohol).

Before data collection, the questionnaire (with all its measurement tools) was piloted in a region outside the study setting (Manhiça district) to ensure its accuracy and effectiveness. Self-reported anxiety and depression were measured using the Hospital Anxiety and Depression Scale (HADS). This is a reliable and valid screening tool widely used among non-psychiatric patients and general populations [45, 46]. It comprises 14 items, seven on anxiety and seven on depressive symptoms. A household head was assumed to have anxiety if they scored 8 or higher on the anxiety sub-scale (HADS-A). Similarly, they were assumed to have depressive symptoms if they scored 8 or higher on the depression sub-scale (HADS-D). For study purposes, a composite psychological health variable was created during data analysis by merging anxiety and depression measures from the HADS. This was done by considering all individuals who either had anxiety or those who had depressive symptoms. This approach aimed to increase the sample size and enhance data interpretation. The combined variable reflects the reality that anxiety and depression often co-occur and offers a more holistic view of psychological distress in the household context.

### Data analysis

All statistical analyses were conducted using Stata statistical software version 18.0 (StataCorp, College Station, TX, USA). The objectives of the study were to investigate the effect of FI on anxiety and depression, and to examine the modifying effect of SEP on this relationship. To achieve this, the study employed propensity score matching, a statistical technique designed to create comparative groups using observational data. In this context, individuals with FI were considered the exposed group, while those without FI constituted the control group. The matching methodology was based on the nearest neighbour technique, with a 1:5 ratio [47].

First, the analysis explored participant characteristics and utilized theory-based logistic regression models to assess bivariate relationships and subsequently identify independent predictors for FS status. Variables were incorporated if they attained a moderate significance level (*p* < 0.25). After the analysis, variables that had a probable association with FS status were incorporated, selecting the model with the optimal Bayes Information Criterion. This model was then employed to compute a propensity score indicating the likelihood of a participant being food insecure.

Propensity score matching was employed to create comparable groups of exposed (food insecure) and unexposed (food secure) participants. Propensity scores were estimated using logistic regression, with FI as the dependent variable and potential confounders as independent variables. The following variables were included in the propensity score model (e.g. socioeconomic and demographic factors).

Matching was performed using 1:5 nearest neighbour matching, where each exposed participant was matched to five unexposed participants with the closest propensity scores. This matching procedure resulted in an analytical sample of 1,174 participants, consisting of 787 exposed and 387 unexposed individuals.

Covariate balance was assessed before and after matching by examining standardized mean differences between the exposed and unexposed groups for each covariate included in the propensity score model. A standardized mean difference of less than 0.1 was considered indicative of good balance.

The average treatment effect on the treated (ATT) was calculated, representing the impact of FI on psychological health outcomes relative to the expected outcomes if the exposed participants had been food secure. Attributable fractions were also calculated to estimate the proportion of psychological ill health attributable to FI among the exposed (AF_exp) and in the total population (AF_pop).

The models included main effects for socioeconomic indicators and FI, as well as their interaction terms:

$$\eqalign{ Psychological \, & health\,\left( {anxiety\,and\,depression} \right) \cr & = \,\beta \_0 \, + \,\beta \_1 \,Income \,level\, + \,\beta \_2\, Work\,status \cr & + \, \beta \_3 \,Education\, level\, + \, \beta \_4 \,Food\, insecurity \cr & + \, \beta \_5\, (Income \,level\, \times \,Food\, insecurity) \cr & + \, \beta \_6 \,\left( {Work\, status\, \times \, Food\, insecurity} \right) \cr & + \, \beta \_7 \,\left( {Education\, level\, \times \, Food\, insecurity} \right)\, + \,\varepsilon \cr}$$

To examine potential effect modification by socioeconomic factors, interaction effects between FI and socioeconomic variables were assessed. We hypothesized that the relationship between FI and psychological health might be modified by SEP, with individuals of higher SEP being less likely to experience poor psychological health when exposed to FI compared to those of lower SEP.

Interaction terms were created by multiplying the binary FI variable with each of the following socioeconomic variables: income level (low, high), work status (paid work, unpaid work), and education level (primary/secondary, high school/university). Stratified analyses were conducted to visualize the patterns of effect modification, with separate regression models fitted for each stratum of the socioeconomic variables. The results of these analyses were presented using interaction plots.

## Results

### Participants’ characteristics

In this study, both HAD sub-scales were internally consistent, with values of Cronbach’s coefficient alpha being 0.83 (HADS-A) and 0.78 (HADS-D) (Supplementary Data 1 and 2). Table 1 shows descriptive statistics for a range of demographic and socioeconomic factors and their association with anxiety and depression. Out of the total sample of 1,842 participants, 1,066 (57.9%) were classified as not having anxiety and depression, while 776 (42.1%) exhibited symptoms consistent with anxiety and depression. Significant disparities were observed regarding FS. The vast majority (79.0%) of participants reported FI, and nearly all of them (99.9%) indicated symptoms of anxiety and depression. This suggests that there is a stark correlation between FI and psychological distress. The distribution across municipal districts was fairly uniform, with the highest representation from KaMaxaquene, at 25.7%, closely followed by KaMubukwana (25.4%). Across the age groups, middle-aged adults represented the majority (57.6%): of these, 59.3% reported symptoms of anxiety and depression. In terms of gender, women were more predominant in the sample, at 63.9%. When evaluating anxiety and depression by gender, a higher percentage of female participants (63.1%) reported mental health symptoms compared to males (36.9%). Median household size and number of children were reported as five and two, respectively, with no differentiation between the two anxiety and depression categories. Regarding marital status, 66.6% of the participants were either married or in a marital union, with 60.6% exhibiting anxiety and depressive symptoms. When analysing food diversity, a large proportion of participants (52.9%) had low food diversity, and a staggering 93.6% of this group showed symptoms of anxiety and depression. Regarding income, the majority (36.3%) were categorized as “low income”, with the vast majority (73.7%) reporting symptoms of psychological ill health. Work type revealed that 50.7% of the sample engaged in paid work, but unpaid workers exhibited higher levels of anxiety and depression (64.7%) compared with paid workers (35.3%). Lastly, concerning education, many participants had achieved high school or university level education (54.6%). Those with only primary or secondary education had a much higher incidence of anxiety and depression, at 75.1%, than did their higher-educated counterparts, at 24.9%.

**Table 1:** Characteristics of participants, Maputo City, southern Mozambique

	Psychological ill health (anxiety and depression)		
	No	Yes	Total
N	1,066 (57.9%)	776 (42.1%)	1,842 (100.0%)
**Food insecurity**			
Food-secure	386 (36.2%)	1 (0.1%)	387 (21.0%)
Food-insecure	680 (63.8%)	775 (99.9%)	1,455 (79.0%)
**Municipal district**			
KaMaxaquene	269 (25.2%)	205 (26.4%)	474 (25.7%)
KaMubukwana	303 (28.4%)	164 (21.1%)	467 (25.4%)
KaMavota	246 (23.1%)	204 (26.3%)	450 (24.4%)
Nlhamankulu	248 (23.3%)	203 (26.2%)	451 (24.5%)
**Age of participant**			
Young adult (< 29 years)	232 (21.8%)	152 (19.6%)	384 (20.8%)
Middle-aged adult (29 − 37)	601 (56.4%)	460 (59.3%)	1,061 (57.6%)
Older adult (> 37)	233 (21.9%)	164 (21.1%)	397 (21.6%)
**Sex of participant**			
Male	379 (35.6%)	286 (36.9%)	665 (36.1%)
Female	687 (64.4%)	490 (63.1%)	1,177 (63.9%)
**Head of household**			
Male	793 (74.4%)	525 (67.7%)	1,318 (71.6%)
Female	273 (25.6%)	251 (32.3%)	524 (28.4%)
Household size	5 (4–5)	5 (4–5)	5 (4–5)
Number of children	2 (1–3)	2 (2–3)	2 (1–3)
**Marital status**			
Single/ separated/ divorced	310 (29.1%)	306 (39.4%)	616 (33.4%)
Married/ marital union	756 (70.9%)	470 (60.6%)	1,226 (66.6%)
**Food diversity**			
Low	249 (23.4%)	726 (93.6%)	975 (52.9%)
Medium	517 (48.5%)	46 (5.9%)	563 (30.6%)
High	300 (28.1%)	4 (0.5%)	304 (16.5%)
**Income**			
Low income	96 (9.0%)	572 (73.7%)	668 (36.3%)
Middle income	459 (43.1%)	190 (24.5%)	649 (35.2%)
High income	511 (47.9%)	14 (1.8%)	525 (28.5%)
**Type of work**			
Unpaid work	406 (38.1%)	502 (64.7%)	908 (49.3%)
Paid work	660 (61.9%)	274 (35.3%)	934 (50.7%)
**Education**			
Primary or secondary	254 (23.8%)	583 (75.1%)	837 (45.4%)
High school or university	812 (76.2%)	193 (24.9%)	1,005 (54.6%)

### Predictive margins of food insecurity against psychological health across age groups

**Fig. 1:**
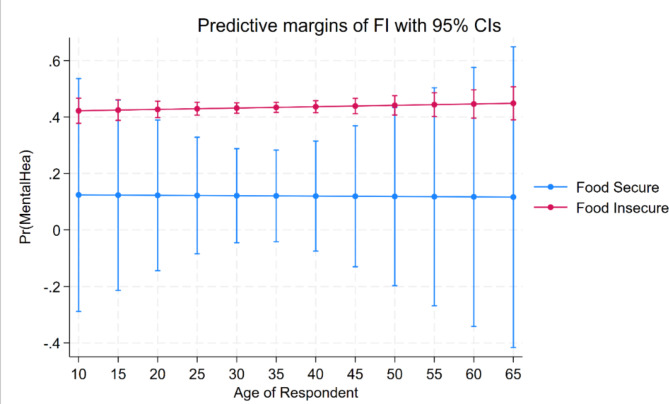
Predictive margins of food insecurity (FI) with 95% confidence intervals (CIs) against the prevalence of anxiety and depression across various age groups of participants

Figure 1 illustrates the predictive margins of FI with a 95% confidence interval (CI) against the prevalence of the psychological health problem (anxiety and depression) by age. Two distinct lines represent the two groups: food-secure (blue) and food-insecure (pink). Across all age groups, those who were food-insecure consistently showed a higher probability of experiencing anxiety and depression compared with their food-secure counterparts. The food-insecure line remains relatively stable around the 0.4 mark, suggesting that around 40% of these individuals were likely to have anxiety and depression, irrespective of age. The 95% CIs (indicated by the vertical bars) for the food-insecure group are narrow, denoting a greater level of reliability in the presented estimates for this group. By contrast, the food-secure group consistently showed a lower probability of having mental health symptoms, remaining below the 0.2 mark, indicating that less than 20% of this group were likely to experience anxiety and depression. However, the 95% CIs for the food-secure group are very wide (particularly at young and older ages), suggesting that the sample size is too small, therefore being less informative (unreliable or uncertain). Anyhow, the prevalence of anxiety and depression among both groups appears to have remained relatively constant, suggesting that within this sample, age does not appear to be a significant factor influencing the likelihood of experiencing mental health problems when considered in the context of FS status.

### Estimation of propensity scores

Propensity scores indicate the likelihood of an individual being in the exposed group based on various covariates. To estimate the propensity scores, we utilized a logistic regression approach. The findings revealed several significant predictors for FI (Table 2). Different municipal districts, namely KaMubukwana, KaMawota, and Nhlamankulu, displayed distinct coefficients, suggesting a variance in level of FI between them. Furthermore, a notable observation was that larger household size and a greater number of children were significant predictors for increased FI. As anticipated, food diversity showed a strong negative association with FI. Also, higher education indicated a reduced likelihood of FI. Finally, there was a clear association between the middle-income and high-income categories and FS.

**Table 2:** Results of logistic regression of the relationship between food insecurity (FI) and socio-demographic characteristics, Maputo City Household Survey, 2022

	Odds ratio	SE	Z	*P*>|z|	95% CI	
Age	0.9981	0.1109	-0.02	0.987	0.8027	1.2411
**Municipal district**						
KaMubukwana	0.4917	0.1057	-3.30	0.001	0.3226	0.7493
KaMavota	1.8964	0.4466	2.72	0.007	1.1953	3.0087
Nlhamankulu	2.0343	0.4706	3.07	0.002	1.2927	3.2011
**Sex**						
Female	0.8478	0.1488	-0.94	0.347	0.6008	1.1961
**Head**						
Female	0.6634	0.2522	-1.08	0.280	0.3148	1.3976
Household size	2.0090	0.2363	5.93	0.000	1.5954	2.5298
Children	1.4894	0.1897	3.13	0.002	1.1602	1.9118
**Marital status**						
Married/ marital union	0.4987	0.1788	-1.94	0.052	0.2470	1.0069
**Food diversity**						
Medium	0.1029	0.0224	-10.45	0.000	0.0672	0.1577
High	0.1043	0.0695	-3.39	0.001	0.0282	0.3848
**Income**						
Middle-income	0.1075	0.0198	-12.07	0.000	0.0748	0.1544
High-income	0.0325	0.0124	-8.98	0.000	0.0154	0.0686
**Work**						
Paid work	1.1463	0.1897	0.83	0.409	0.8287	1.5856
**Education**						
High school or university	0.6907	0.1171	-2.18	0.029	0.4954	0.9629
_cons	0.0120	0.0465	-1.14	0.254	6.06e-06	23.7853

### Covariate balancing test

**Fig. 2:**
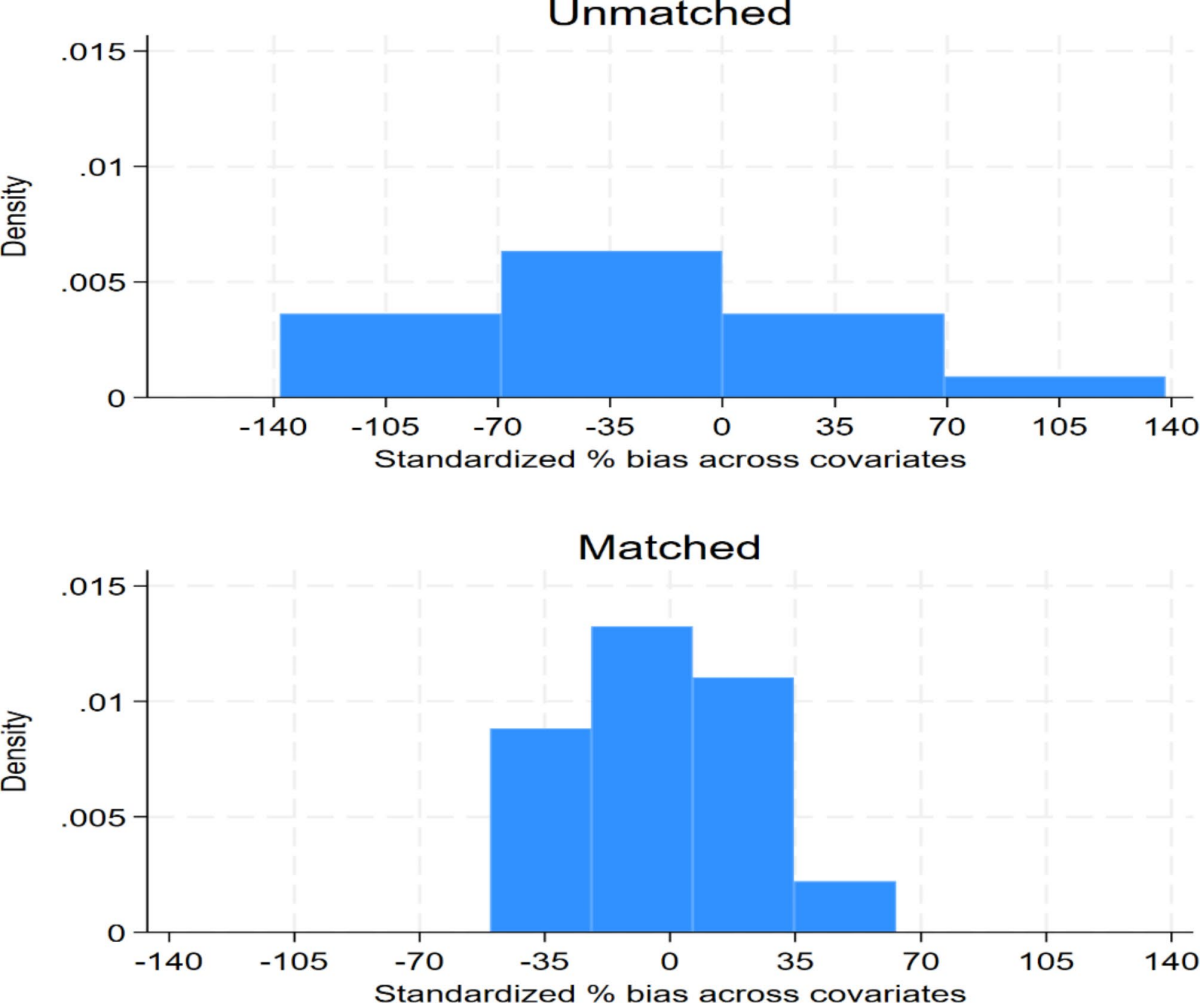
Standardized percentage bias across covariates for matched and unmatched samples

Figure 2 compares the distribution of standardized percentage bias across covariates for matched and unmatched data. As shown in the Figure, the distribution of the standardized percentage bias across covariates for the unmatched data is somewhat centred around zero but there is a notable spread, with biases extending beyond − 140% to the left and 140% to the right. There are more covariates with positive biases than there are covariates with negative biases. This indicates that, prior to matching, there may be a systematic overrepresentation of certain covariates in the exposed group compared with the control group. However, post-matching, the distribution of biases is tighter and more centred around zero; while there are still some biases, their magnitude is reduced compared with the unmatched data. The distribution after matching appears to be slightly skewed to the right, indicating that there are a few covariates for which the exposed group still has a slight overrepresentation compared with the control group. However, this skewness is significantly less pronounced than in the unmatched data. The propensity score matching process has effectively reduced the bias in the covariates between the exposed and control groups. This is evident from the comparison of the two distributions where the matched distribution is more concentrated around zero, indicating improved balance across the covariates.

### Association between food insecurity and psychological health

Of the 1,842 participants in the original sample, 787 food-insecure participants were matched with 387 food-secure participants using propensity score matching (ratio 1:5). This reduction in sample size after matching is expected, as participants without a suitable match are discarded in the matching process to ensure comparability between the exposed and unexposed groups.

The propensity score matching analysis revealed a substantial difference in the risk of poor psychological health between the exposed and unexposed groups (Table 3). The risk of poor psychological health among participants exposed to FI was 25.79%, compared to only 0.26% among those not exposed. The risk difference was 25.54% points (95% CI: 22.44–28.63), with a risk ratio of 99.82 (95% CI: 14.05–709.40, *p* < 0.001). The attributable fraction among the exposed (AF_exp) was 98.99%, indicating that almost all the risk for poor psychological health in the exposed group could be attributed to FI. The population attributable fraction (AF_pop) showed that 98.51% of poor psychological health in the total population could be attributed to FI. This trend was evident in the descriptive analysis, as the heads of households suffering from moderate and severe FI exhibited higher scores on both anxiety and depression subscales (7.83 [95% CI: 7.75 − 7.91] vs. 6.94 [95% CI: 6.82 − 7.07]) compared to their food-secure and mild food-insecure counterparts (4.65 [95% CI: 4.56 − 4.74] vs. 4.59 [95% CI: 4.51 − 4.68]), respectively.

**Table 3:**
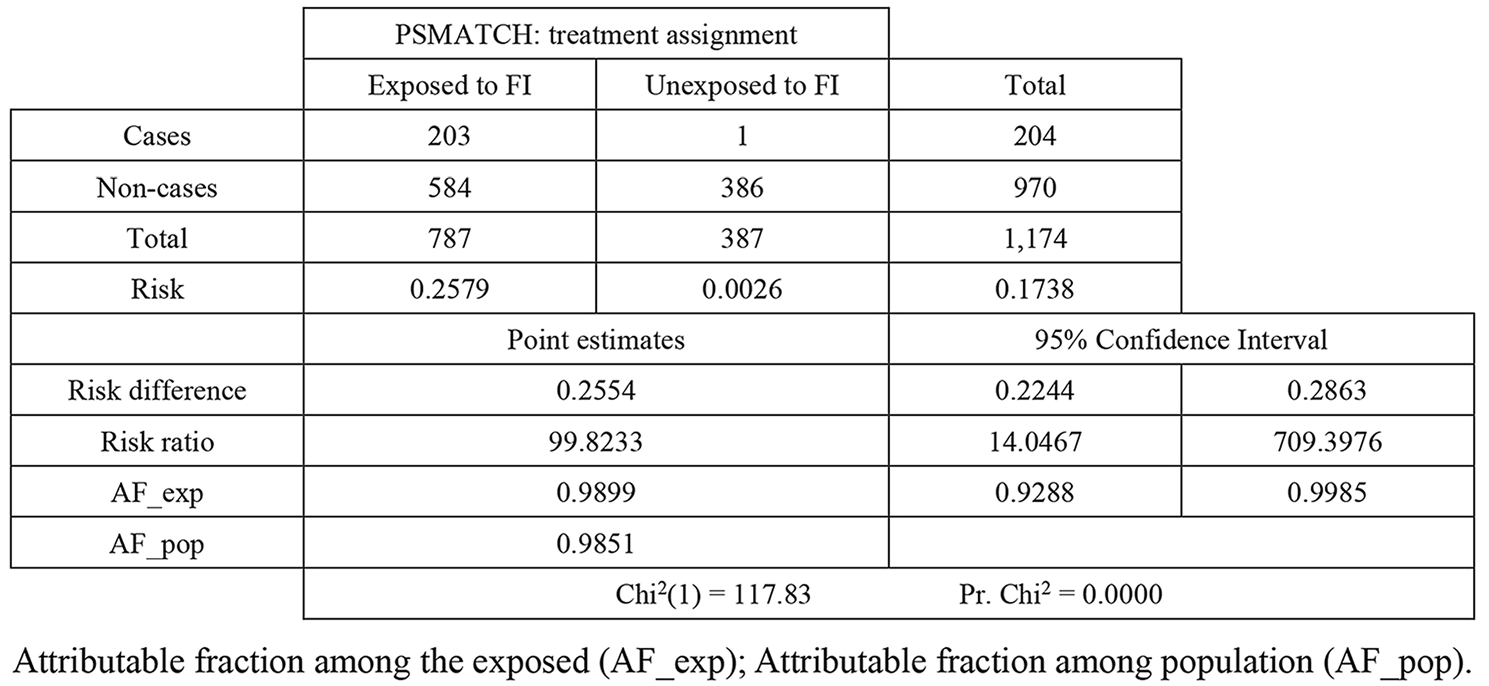
Association between food security (FS) status and psychological health outcomes (anxiety and depression)

### Interaction effects

**Fig. 3:**
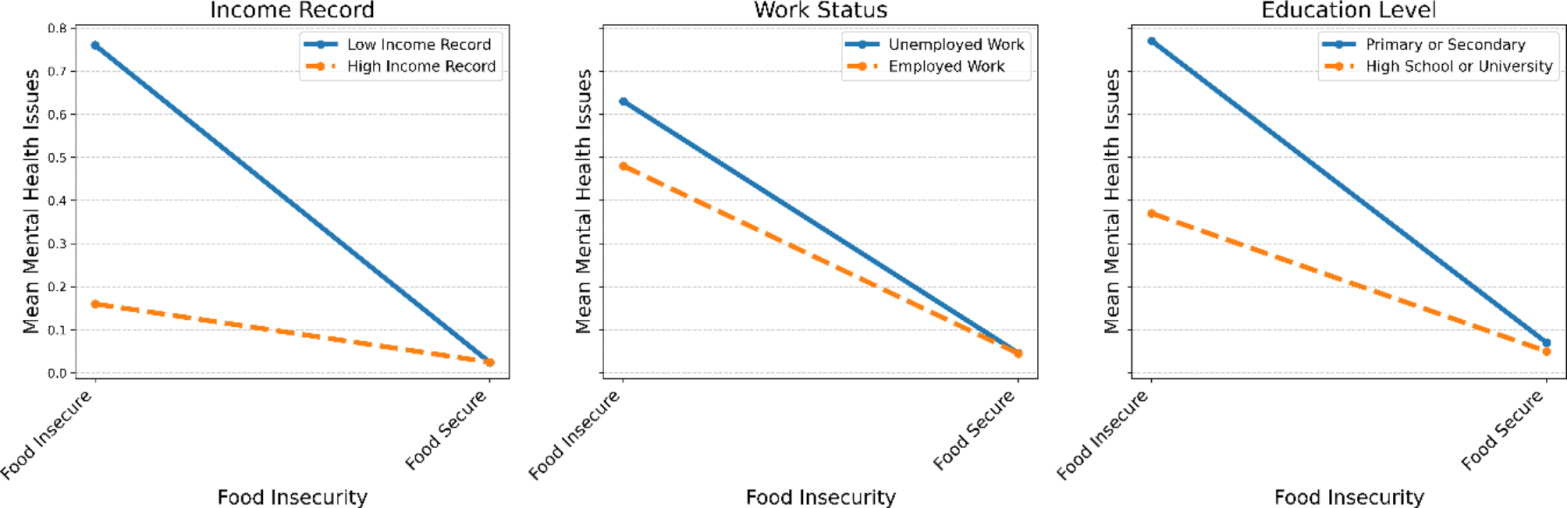
Interaction effects between food insecurity (FI) and the socioeconomic position (SEP) of the household head, and psychological health (anxiety and depression)

The interaction effects analysis revealed that the association between FI and psychological health was modified by the SEP of the household head (Fig. 3). Heads of food-secure households with higher incomes appeared to have better psychological health outcomes compared to their food-insecure counterparts with low income. Similarly, heads of food-secure households with paid work tended to report better psychological health compared to their food-insecure counterparts with unpaid work. This pattern was also observed for education level, with food-secure participants with higher education levels reporting better psychological health compared to their food-insecure counterparts with primary or secondary education. These findings suggest that socioeconomic factors, particularly income and education, may buffer the negative impact of FI on psychological health. Household heads with a higher SEP seem to be less likely to experience poor mental health outcomes when exposed to FI compared to those with a lower position.

## Discussion

In the present study, propensity score matching was utilized to explore the potential association between FI and psychological health. Propensity score matching is an innovative and advanced approach that has been used to assess causality in observational studies where it is ethically challenging or impractical to perform randomized controlled trials [47, 48].

The sample randomly assigned to propensity score matching consisted of 1,174 participants, 787 of whom were exposed and 387 of whom were unexposed to FI. A marked disparity between the two groups was evident in terms of adverse psychological health. The exposed group demonstrated a risk of 25.79%, while the unexposed group’s risk was a mere 0.26%, indicating a notably higher likelihood of adverse psychological health outcomes among those who experienced FI.

The risk difference, representing the absolute change in risk between the exposed and unexposed groups, was substantial, 0.2554. The 95% confidence interval (CI) for this metric ranged from 0.2244 to 0.2863, suggesting a high level of statistical confidence in the observed risk difference. In relative terms, the risk ratio was a staggering 99.82, indicating that the risk for adverse psychological health outcomes was nearly 100 times higher among those exposed compared with those unexposed to FI. The attributable fraction values (among the exposed population and the entire population) underscore the magnitude of the potential impact of FS on psychological health at both the individual and the population level. Anyhow, it is important to recognize that there is a strong stigma attached to mental health conditions in Mozambique, that most people choose to suffer in silence rather than admitting their struggles with mental health issues [49, 50]. This sad reality was noticeable looking at the HADS scores, as the heads of households suffering from moderate and severe FI displayed higher scores on both anxiety and depression subscales compared to their food-secure and mild food-insecure counterparts who displayed lower scores. Other sociodemographic factors presented in Table 1 confirmed inequalities in mental health between groups. This situation reinforces the need to raise public awareness about mental health in the country. Similar results pointing to increased psychological ill health among food-insecure households compared with their food-secure counterparts have been found across the world [15, 51], in the USA [52], Canada [53], France [54], Brazil [19], Mexico [55] and Taiwan [56], and across Africa [33].

Likewise, this study reveals that FS serves as a significant buffer in the context of socioeconomic adversity. Households with paid work reported better psychological health when FS was assured, highlighting the compound burden of unpaid work and FI. Income level, a strong socioeconomic indicator, interacts with FS status, where being in a higher income bracket has a protective effect against psychological health decline in the context of FS. Education level further compounds these effects, suggesting that higher educational attainment in conjunction with FS is associated with more favourable psychological health outcomes. These findings underscore the intricate interplay between FS and SEP, suggesting that interventions aimed at improving FS may have beneficial implications for psychological health across a range of socioeconomic scenarios. The interaction analyses showed that higher levels of FI are associated with poorer psychological health status across all SEPs. This is consistently shown by the upward trend in psychological health scores in the food-insecure groups compared with the food-secure groups, regardless of SEP. Being in a food-secure household seems to buffer against poor psychological health outcomes to some degree, while FI exacerbates psychological ill health.

In agreement with our findings, a few studies conducted outside Africa have shown the significance of SEP to modify the relationship between FI and psychological health, and they have suggested FS strategies as practical ways to improve psychological health among vulnerable populations [36–38]. On the other hand, various studies have illustrated the importance of socioeconomic factors (e.g. education, work and income) on household FI [26, 57, 58] and psychological health [4, 27].

Socioeconomic inequalities are globally recognized as the most fundamental causes of health inequalities [59, 60] and are among the underlying causes of poverty and household FI, notably in the LMICs [26]. It is well established that each factor that constitutes SEP has its impact on household FI [26] and psychological health [28], just as observed in this study. For instance, education can shape a person’s career opportunities, as well as provide knowledge and skills that allow better-educated people to access more information and resources to promote their health [61]. At the same time, education can greatly influence food access and utilization [29].

Similarly, employed people, compared with unemployed people, are more likely to have better mental health outcomes. Higher incomes may provide the means for better housing, schooling, health care, nutrition and recreation [28, 61]. Furthermore, chronic stress attached to lower SEP can increase morbidity and mortality [59, 62]. In parallel, poverty and low income derived from various factors [63, 64] can prevent vulnerable individuals and households from having a quality diet [1], adequate housing, and quality education for their families [28]. A lack of employment and even underemployment of heads of households can push their families into poverty and household FI, thereby contributing to and worsening psychological health [30]. Furthermore, financial difficulties can reduce people’s sense of agency, control and self-esteem, contributing to poorer psychological health [38].

At the same time, SEP underlies other key determinants of health, such as health care, environmental exposure, and health behaviours [61, 65]. In this context, higher positions hold advantages in accessing resources, information and circumstances that are more conducive to better mental health [61, 66] and to ensuring FS among households. Therefore, reducing inequalities in SEP and household FI will require coordinated and joint efforts from various actors and institutions that effectively address each of these components as well as the pathways by which they affect people’s mental health [59].

Altogether, the findings of this study suggest that various factors (e.g. demographic, and socioeconomic factors) have a significant relationship with household FI and psychological health. Household FI appears to be a stressful condition in itself that has an independent and significant impact on psychological health. In fact, household FI has an extensive negative effect on various psychological health outcomes, among which, notably higher levels of FI are associated with a higher risk of adverse mental health [52, 67]. Several studies conducted in LMICs and HICs point out that FI and psychological ill health are related in a vicious cycle [33, 68]. Nonetheless, as noted in the present study, the strength of this relationship depends heavily on the severity of the FI itself [4], and on various other factors that include the characteristics of the population studied (socioeconomic and demographic factors), the measuring instruments used and the time period for data collection [69–71].

To conclude, the statistical significance of the observed associations in this study (Chi^2^ = 117.83, *p* = 0.0000) emphasizes the robustness of the findings and the unlikelihood of random chance. It is evident that FI is a public health concern and a modifiable determinant of health worthy of macro-level policy intervention [53]. Therefore, public health policies and intervention programmes aimed at alleviating household FI and promoting decent work and quality education, as well as other livelihood initiatives, could be much more effective in reducing the burden of psychological illness among vulnerable populations if implemented together as a whole.

### Strengths and limitations

This is one of the few quantitative studies that provide recent empirical evidence on the relationship between SEP of the household head, household FI and psychological ill-health (anxiety and depression) in Maputo City in the context of the COVID-19 pandemic. The propensity score matching process was successful in creating comparable groups and was effective in reducing the bias in the covariates between the exposed and the control group. The study used validated measures of household FI and psychological health outcomes. A considerable number of household heads participated in the study, and the findings can be generalized to the entire city of Maputo.

Nonetheless, some limitations must be considered. A cross-sectional analysis such as this does not show causality; however, using propensity score matching, household FI clearly stands out as a key modifiable determinant of psychological health among vulnerable populations. It is also important to consider the potential for residual confounding and bias due to not including an important factor in the calculation of a propensity, especially when data on these factors have not been collected [47, 48]. At the same time, the predictor and outcome measures were based on self-report, which may be subject to response bias. Similarly, although FI was measured in all its levels (FS, and mild, moderate, and severe FI), the sample size was too small to explore this powerful information through multilevel analysis. In fact, just as in this study, it has been shown globally that the impact of household FI on psychological health largely depends on the severity of household FI attached to various structural factors (e.g. income, employment, education) [15, 51].

Regarding the interpretation of the burden of psychological ill health observed in this study, this requires some caution. The instrument used, the HADS, is a screening tool for identifying and quantifying anxiety and depression. Because of the presence of a strong general distress factor, the HADS does not provide good separation between symptoms of anxiety and symptoms of depression. Therefore, it is best used as a measure of general psychological distress, and for identifying general hospital patients, as well as members of the general population, who need further psychiatric evaluation and assistance [45, 46]. Furthermore, considering the strong stigma historically attached to mental health in Mozambique [49, 50] and recognizing that mental health exists in a complex continuum [72], additional caution is required when interpreting our findings.

Likewise, more than usual caution is warranted when interpreting the burden of psychological ill health considering that the data collection was conducted just after the COVID-19 pandemic. For instance, a systematic review by Nochaiwong et al. [73] reported a global prevalence, during the COVID-19 pandemic, of psychological ill health among the general population of 28.0% for depression; 26.9% for anxiety; 36.5% for stress; 50.0% for psychological distress; and 27.6% for sleep problems [73]. Unfortunately, the authors did not include the proportion of each mental condition attributable to household FI. Nonetheless, there is evidence suggesting that household FI may be a relevant contributor to all of these adverse health conditions [74, 75].

Finally, future research should examine the above-mentioned relationships longitudinally (e.g. prospective cohort studies and ethnographic studies) to gain a deeper and more exhaustive understanding of the mechanisms behind these associations (household FI, socioeconomic and demographic factors, and psychological health) and to establish causal inferences.

## Conclusions

The findings of this study provide evidence for the relationship between FI and anxiety and depression. In addition, the results show that the SEP of household heads (particularly regarding income and education) appears to greatly modify the relationship between household FI and psychological ill health.

Addressing inequalities in household FI, along with SEP, could therefore be a key strategy in promoting better psychological health in the studied population. Specifically, as a short-term solution the findings demand urgent informed actions to provide social and economic support (e.g. cash transfers, basic food basket assistance) to the most vulnerable groups (including households headed by individuals with a lower SEP). Similarly, targeted policies and intervention programmes aimed to alleviate FI at all levels (including local and regional) and promote quality education and decent work and improve livelihoods among the most vulnerable groups are warranted.

## Electronic supplementary material

Below is the link to the electronic supplementary material.

Supplementary Material 1

## Data Availability

The data presented in this paper are not publicly available owing to restrictions in the ethical approval for this study. However, de-identified data can be made available by the corresponding author on reasonable request.

## Abbreviations

AFexp: Attributable fraction among the exposed
AFpop: Attributable fraction among the population
CI: Confidence interval
DALYs: Disability-adjusted life years
FI: Food insecurity; FS: food security
HADS: Hospital Anxiety and Depression Scale
HADS-A: Hospital Anxiety and Depression Scale anxiety sub-scale
HADS-D: Hospital Anxiety and Depression Scale depression sub-scale
HICs: High-income countries
LMICs: Low- and middle-income countries
PSMATCH: Propensity score matching
SE: Standard error
SEP: Socioeconomic position
SETSAN: Mozambique Technical Secretariat for Food Security and Nutrition
YLDs: Years lived with disability
