# Supplementary material for: Association between socioeconomic position of the household head, food insecurity and psychological health: an application of propensity score matching
Source: BMC Public Health. 2024 Sep 27;24:2590. doi: 10.1186/s12889-024-20153-0 (PMC11429249; doi:10.1186/s12889-024-20153-0)
Supplement: Supplementary file 1 — Supplementary Material 1 [file 12889_2024_20153_MOESM1_ESM.docx]

**Supplementary material**

**Supplementary Data 1. Reliability of the anxiety sub-scale (HADS-A).**

| **Reliability Statistics** | | |
| --- | --- | --- |
| Cronbach's Alpha | Cronbach's Alpha Based on Standardized Items | N of Items |
| .830 | .852 | 7 |

| **Inter-Item Correlation Matrix** | | | | | | | |
| --- | --- | --- | --- | --- | --- | --- | --- |
|  | Item 1 | Item 3 | Item 5 | Item 7 | Item 9 | Item 11 | Item 13 |
| Item 1 | 1.000 | .186 | .456 | .447 | .521 | .377 | .350 |
| Item 3 | .186 | 1.000 | .277 | .404 | .453 | .359 | .351 |
| Item 5 | .456 | .277 | 1.000 | .580 | .681 | .303 | .658 |
| Item 7 | .447 | .404 | .580 | 1.000 | .512 | .437 | .654 |
| Item 9 | .521 | .453 | .681 | .512 | 1.000 | .479 | .651 |
| Item 11 | .377 | .359 | .303 | .437 | .479 | 1.000 | .337 |
| Item 13 | .350 | .351 | .658 | .654 | .651 | .337 | 1.000 |

| **Summary Item Statistics** | | | | | | | |
| --- | --- | --- | --- | --- | --- | --- | --- |
|  | Mean | Minimum | Maximum | Range | Maximum / Minimum | Variance | N of Items |
| Inter-Item Correlations | .451 | .186 | .681 | .495 | 3.665 | .018 | 7 |

| **Item-Total Statistics** | | | | | |
| --- | --- | --- | --- | --- | --- |
|  | Scale Mean if Item Deleted | Scale Variance if Item Deleted | Corrected Item-Total Correlation | Squared Multiple Correlation | Cronbach's Alpha if Item Deleted |
| Item 1 | 7.1268 | 10.855 | .529 | .356 | .822 |
| Item 3 | 7.7465 | 12.735 | .445 | .284 | .826 |
| Item 5 | 6.9155 | 12.793 | .687 | .585 | .804 |
| Item 7 | 7.5070 | 10.054 | .673 | .556 | .792 |
| Item 9 | 6.9155 | 9.678 | .745 | .656 | .776 |
| Item 11 | 7.8732 | 12.884 | .527 | .320 | .817 |
| Item 13 | 7.0423 | 12.727 | .689 | .602 | .803 |

| **Scale Statistics** | | | |
| --- | --- | --- | --- |
| Mean | Variance | Std. Deviation | N of Items |
| 8.5211 | 15.453 | 3.93105 | 7 |

**Supplementary Data 2. Reliability of the depression sub-scale (HADS-D).**

| **Reliability Statistics** | | |
| --- | --- | --- |
| Cronbach's Alpha | Cronbach's Alpha Based on Standardized Items | N of Items |
| .779 | .769 | 7 |

| **Inter-Item Correlation Matrix** | | | | | | | |
| --- | --- | --- | --- | --- | --- | --- | --- |
|  | Item 2 | Item 4 | Item 6 | Item 8 | Item 10 | Item 12 | Item 14 |
| Item 2 | 1.000 | .287 | .064 | .309 | .234 | .090 | .349 |
| Item 4 | .287 | 1.000 | .209 | .323 | .434 | .349 | .371 |
| Item 6 | .064 | .209 | 1.000 | .441 | .511 | .079 | .216 |
| Item 8 | .309 | .323 | .441 | 1.000 | .609 | .267 | .371 |
| Item 10 | .234 | .434 | .511 | .609 | 1.000 | .273 | .615 |
| Item 12 | .090 | .349 | .079 | .267 | .273 | 1.000 | .372 |
| Item 14 | .349 | .371 | .216 | .371 | .615 | .372 | 1.000 |

| **Summary Item Statistics** | | | | | | | |
| --- | --- | --- | --- | --- | --- | --- | --- |
|  | Mean | Minimum | Maximum | Range | Maximum / Minimum | Variance | N of Items |
| Inter-Item Correlations | .322 | .064 | .615 | .551 | 9.648 | .022 | 7 |

| **Item-Total Statistics** | | | | | |
| --- | --- | --- | --- | --- | --- |
|  | Scale Mean if Item Deleted | Scale Variance if Item Deleted | Corrected Item-Total Correlation | Squared Multiple Correlation | Cronbach's Alpha if Item Deleted |
| Item 2 | 5.0704 | 9.295 | .332 | .209 | .780 |
| Item 4 | 5.4085 | 8.502 | .498 | .281 | .754 |
| Item 6 | 5.4648 | 8.852 | .400 | .311 | .770 |
| Item 8 | 5.0141 | 7.757 | .605 | .443 | .731 |
| Item 10 | 5.1549 | 6.276 | .728 | .625 | .697 |
| Item 12 | 5.0845 | 8.936 | .361 | .219 | .776 |
| Item 14 | 5.3099 | 7.017 | .605 | .476 | .730 |

| **Scale Statistics** | | | |
| --- | --- | --- | --- |
| Mean | Variance | Std. Deviation | N of Items |
| 6.0845 | 10.621 | 3.25904 | 7 |
